# Analytical Methods for the Determination of Pharmaceuticals and Personal Care Products in Solid and Liquid Environmental Matrices: A Review

**DOI:** 10.3390/molecules29163900

**Published:** 2024-08-17

**Authors:** Abdulmalik M. Alqarni

**Affiliations:** Department of Pharmaceutical Chemistry, College of Clinical Pharmacy, Imam Abdulrahman Bin Faisal University, King Faisal Road, P.O. Box 1982, Dammam 31441, Saudi Arabia; amalqarni@iau.edu.sa

**Keywords:** PPCPs, WWTPs, STP, persistence, sample preparation, instrumental analysis, matrix effect, clean-up, analytical challenges

## Abstract

Among the various compounds regarded as emerging contaminants (ECs), pharmaceuticals and personal care products (PPCPs) are of particular concern. Their continuous release into the environment has a negative global impact on human life. This review summarizes the sources, occurrence, persistence, consequences of exposure, and toxicity of PPCPs, and evaluates the various analytical methods used in the identification and quantification of PPCPs in a variety of solid and liquid environmental matrices. The current techniques of choice for the analysis of PPCPs are state-of-the-art liquid chromatography coupled to mass spectrometry (LC-MS) or tandem mass spectrometry (LC-MS^2^). However, the complexity of the environmental matrices and the trace levels of micropollutants necessitate the use of advanced sample treatments before these instrumental analyses. Solid-phase extraction (SPE) with different sorbents is now the predominant method used for the extraction of PPCPs from environmental samples. This review also addresses the ongoing analytical method challenges, including sample clean-up and matrix effects, focusing on the occurrence, sample preparation, and analytical methods presently available for the determination of environmental residues of PPCPs. Continuous development of innovative analytical methods is essential for overcoming existing limitations and ensuring the consistency and diversity of analytical methods used in investigations of environmental multi-class compounds.

## 1. Introduction

Pharmaceuticals and personal care products (PPCPs) fall into numerous chemical classes and are widely used in industry, medicine, and daily life, making them integral components of modern society. Pharmaceuticals, including antibiotics, hormones, analgesics, antiepileptics, β-blockers, and cytostatic drugs, are mainly used for treating or preventing human and animal diseases, whereas personal care products, such as preservatives, fragrances, sunscreens, and disinfectants, improve the quality of daily life [1]. The diversity of PPCPs means that they also have diverse adverse effects, including potential accumulation and environmental issues based on their physiochemical properties (Table 1). Therefore, they have been receiving increasing environmental attention as emerging contaminants (ECs) [2].

Over the past few years, scientific and regulatory organizations have begun to address the effects and risks of PPCPs as potentially bioactive environmental contaminants. As ECs, PPCPs can be classified as groups of chemical materials that hypothetically pose a threat to the environment [3]. In general, ECs include any compounds produced naturally or synthetically for use in everyday products, such as pesticides, cleaning materials, and agricultural products, and they can persist in the environment following their release [4]. At the present time, the lack of regulations for controlling ECs has prompted some attempts in the EU and the US to generate lists of priority contaminants and control their environmental release. For instance, several strategies have been developed in Canada and Switzerland to reduce the release of ECs [5,6,7]. In the US, only a few of the millions of EC materials have been documented and regulated by the US Environmental Protection Agency (US EPA) [3,8,9], as the adverse effects are considered small or negligible, since their concentrations are very low, suggesting no need for control. However, the chronic and translated toxicity of ECs over generations, the growing world population, and the increasing release rate have made ECs, including PPCPs, materials of great concern in the scientific community. 

Although only trace concentrations of PPCPs are typically detected in the environment, the effects of these chemicals in terms of microbial resistance, chemical persistence, and synergistic effects, even at these low levels, are unknown [10]. The presence and persistence of PPCPs in different matrices varies depending on different factors [11], including the sources of different chemicals (e.g., wastewater treatment plants, hospitals, and industries) [12]. Thus, meeting the challenge of PPCP analysis in complex environmental sample matrices requires specific analytical methods and techniques that can assess minute concentrations of these chemicals and their degradation products [13]. 

The recent literature shows a notable increase in the publication of validated analytical methods for the determination of different PPCPs in various environmental matrices, including water samples (e.g., wastewater, surface water, and groundwater), sediments and soils, sludges, and plants. However, most of the recent work was performed on a limited number of analytes in a specific matrix. Thus, the aim of the present work is to provide an overview of recent developments in sample preparation and instrumental analysis of the major PPCPs found in environmental solid and liquid samples. The review pays particular attention to the possible challenges that must be overcome during the analysis of PPCPs in environmental matrixes and highlights the importance of developing environmentally friendly methods that adhere to the principle of green analytical chemistry. 

## 2. Environmental Risks Posed by PPCPs

### 2.1. Sources, Occurrence, and Persistence of PPCPs

**Figure 1:**
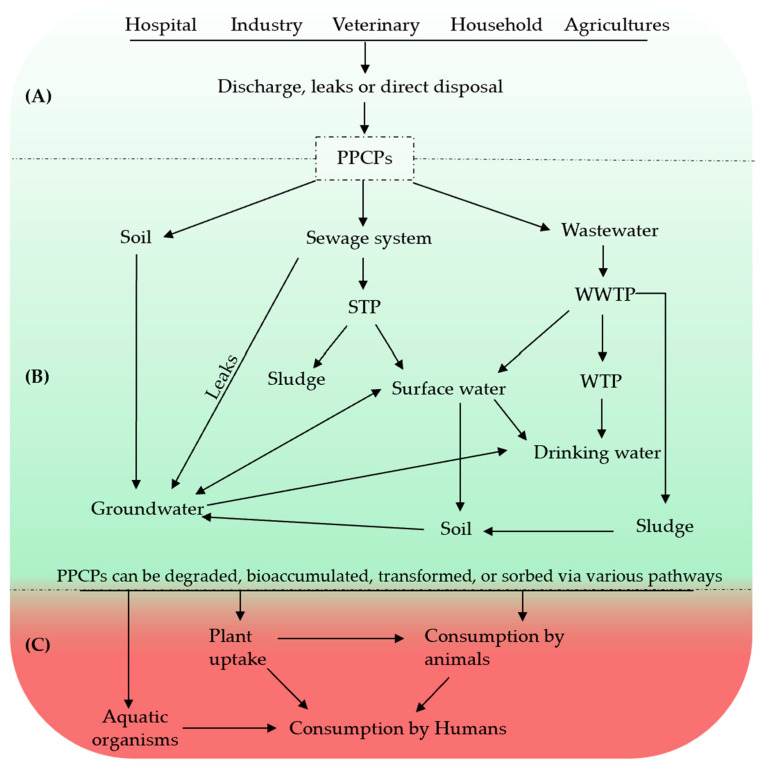
Illustration of (**A**) source, (**B**) circulation (green), and (**C**) exposure (red) of environmental systems with PPCPs. STP: sewage treatment plant; WWTP: wastewater treatment plant; WTP; water treatment plant.

PPCPs are released into the environment via different routes, including release during their manufacture and use in human or animal medicine, and discharge from factories, households, healthcare facilities, farms, and veterinary clinics (Figure 1A) [3]. The main sources of PPCP infusion into the environment are landfill leachates, sewage treatment plants (STPs), and wastewater treatment plants (WWTPs) (Figure 1B) [14]. Present-day wastewater treatment methods are inefficient and do not completely eradicate PPCPs, meaning that water bodies receiving discharges from STPs and WWTPs can be contaminated by the PPCPs present in sewage [12,15]. These materials can also migrate into sewage sludge [16]. For example, in China, antibiotics are considered the most abundant group of pharmaceuticals encountered in STPs [17]. PPCPs in the environment can undergo conversion processes (either total or partial) to produce metabolites with known or unknown toxicities [18] and unknown removal efficiencies by water treatment processes [19]. 

In addition, some PPCPs are not necessarily persistent and have short lifetimes, but their continuous consumption and release into the environment grant them a ‘pseudo-persistent’ status [14,20]. These materials may exist in greater volumes than other organic contaminants, such as pesticides, because of their continuous release and conversion after degradation. Based on their dissipation times, several pharmaceuticals and their metabolites have been categorized as low-, moderate-, and high-persistence compounds in sediments and water systems. Some prevalent pharmaceutical agents classified as low-persistence compounds include paracetamol, ibuprofen, 2-hydroxyibuprofen, and CBZ-diol, while others, such as ivermectin, oxazepam, and iopromide, are classified as moderately persistent, and carbamazepine, diazepam, and clofibric acid are designated as highly persistent chemicals [21,22]. Another study classified oxazepam as having long-term persistence in lake water [23]. 

In agricultural farmlands, the treated WWTP sludge used as fertilizer serves as another source of PPCP contamination. The use of animal dung contaminated with PPCPs can similarly introduce these chemicals into the soil (Figure 1B). Leaching of PPCPs from the soil into groundwater can then contaminate drinking and fresh water reserves [24]. Several PPCPs have been reported in different matrices, including surface water, groundwater, sediments, and soil [11]. Thus, human exposure to PPCPs from different sources, even at low concentrations, can be significant and continuous, and the presence of antibiotics and antimicrobials in the environment may be an important contributor to present-day antibiotic resistance [25,26]. 

Several studies have reported the accumulation of PPCPs and their related metabolites in marine ecosystems [27,28] and other aquatic environments [21,25,29,30]. The occurrence of PPCPs in a given environment varies in different countries and between different regions in the same country, depending on the PPCP consumption and utilization in the region under study. However, these differences make meaningful global comparison of PPCP levels impossible. The occurrence and concentration of some classes of PPCPs reported in different matrices are presented in Table 2. 

### 2.2. PPCP Exposure and Toxicity

Environmental exposure to PPCPs may accelerate their potential harm and effects on ecosystems and human health, as shown in Figure 1C. Pharmaceuticals are designed to target a specific organism; however, they can have biological activities in nontarget organisms that are exposed to chronic trace levels of ECs. In general, chronic exposure to trace levels of a pharmaceutical, particularly at certain sensitive stages, can cause more harmful effects than are observed following acute high-dose exposure [50,51]. Potential ecotoxicities were explored by Jjemba [52] to assess the potency of PPCPs based on bioavailability, duration of exposure to nontarget organisms, and chronic toxicity. The take-home message was that studies on the impacts of pharmaceuticals present in the environment need to consider the potential effects of exposure and bioaccumulation of these compounds in biota.

Several groups have conducted ecotoxicology and risk assessment studies on different pharmaceutical compounds [53,54,55], but personal care products have received significantly less attention. However, the extensive use of personal care products, their inappropriate disposal in surface waters and wastewaters, and their ineffective remediation methods have made these products a major source of toxicity in many ecosystems [17,51,56,57]. Many personal care chemicals can act as endocrine-disrupting agents with acute and chronic toxicity in aquatic species [58]. The types of endocrine-disrupting personal care products include certain disinfectants, fragrances (e.g., musk xylol, galaxolide, and celestolide), and UV screens (e.g., camphor and benzophenone-3) [21]. Triclocarbon shows toxicity and enhances the mortality of biofilm algae and aquatic bacteria [59]. Parabens (PBs), used as preservatives in cosmetics, have been reported to show acute toxicity, which is positively correlated with their alkyl chain length; thus, methyl PB and ethyl PB are less toxic than propyl PB and butyl PB toward invertebrates and fish [60]. UV filters employed in sunscreens, lotions, and cosmetics have the ability to cause estrogenic effects and to disrupt the endocrine systems of fishes [51,61].

Many pharmaceuticals, including glucocorticoids, hormones, veterinary growth hormones, and non-steroidal pharmaceuticals, are also endocrine disruptors [21], and the PPCP mixtures encountered in environmental matrices can have synergistic effects with considerable ecotoxicity [62]. For instance, synergistic effects were reported for carbamazepine and clofibric acid, which showed stronger effects on *Daphnia magna* when tested in combination compared to either drug alone [63]. Diclofenac was shown to affect the kidneys and different immune signaling pathways in brown trout [64]. Propranolol, a selected β-blocker, was considered harmful to aquatic organisms [65] and affected the reproduction of *Ceriodaphnia dubia* and *Hyalella azteca* [66]. Several studies have shown that naproxen bioaccumulates in aquatic ecosystems and induces oxidative stress, genetic disruption, and growth inhibition in aquatic organisms [67]. Methotrexate, an anticancer drug, caused acute toxicity in *Tetrahymena pyriformis* and teratogenicity in fish embryos [68,69].

The presence of PPCPs in the environment raises another important concern regarding antibiotic resistance in different organisms. In Australia, several antibiotics detected in the effluent of a WWTP were reported to cause increased resistance of two natural bacterial strains present in the receiving water [70]. Another study reported the toxicity of ciprofloxacin to green algae [71], the toxicity of many fluoroquinolone antibiotics to different aquatic organisms, and the toxicity of oxolinic acid to *D. magna* [72]. Another study elucidated the major source of antibiotic resistance [26]. Overall, the control strategies used to characterize and overcome resistance developing from PPCP contaminants should incorporate advanced biotechnologies. Considering the extensive usage of PPCPs, only a small proportion is actually tested, quantified, and risk assessed in different matrices. The evaluation of PPCP occurrence and toxicity requires fast and precise analytical techniques under optimum conditions to allow their detection at the low concentration levels (i.e., ng/L) typically encountered in environmental samples.

## 3. Sample Preparation, Extraction, and Clean-Up

The design of reliable and validated methods to identify and quantify PCPPs as ECs is currently one of the major obstacles to successful analytical methodology. Sample pretreatments, including extraction and isolation of the analytes of interest from the sample matrices, are required before analytical instrumentation applications (Table 3 and Table 4). Compound isolation from different matrices should be performed with minimal amounts of solvents, optimal extraction efficiency, and high recovery with good reproducibility [73]. The recent use of green solvents in different extraction techniques has been assessed in several studies to address greenness profiles [39,73,74,75,76,77,78]. Analysis typically requires many primary treatment stages, including pH changes, filtration, centrifugation, separation, and sample concentration. Several sample matrices, such as sewage sludge, must undergo pretreatment to facilitate extraction and reduce matrix effects before instrument detection. The published methods report an endless number of extraction or modified extraction techniques from different matrices.

### 3.1. Solid-Phase Extraction (SPE)

Many extraction procedures have been published for trace analysis of PPCPs as ECs in different matrices. As shown in Table 3 and Table 4, SPE with various sorbents is the most routinely applied extraction method. The SPE technique typically utilizes a small amount of sorbent contained in a cartridge or syringe barrel. After activating the sorbent, the sample is passed through it, and the analyte of interest is retained. The retained analyte is usually eluted by passing through a few mL of the appropriate solvent. The commercially available sorbents work primarily with two different separation mechanisms: reversed phase chromatography (RP) and ion exchange chromatography (IC). PPCPs, including acidic, basic, and neutral compounds with reasonable hydrophobicity, can easily be extracted by SPE using RP materials, such as poly(styrene-divinylbenzene) or alkyl-modified silica. However, the extraction of environmental samples containing pharmaceuticals with quite polar properties is more difficult using traditional RP materials. Thus, mixed-mode materials, such as Oasis HLB, with dual polarities, are gaining in popularity. Oasis HLB is a polymer of divinylbenzene and vinylpyrrolidone and is the most widely utilized sorbent because of its hydrophilic–lipophilic balance and wide range of polarities and pH values [99]. Other materials, such as Oasis MCX and Oasis WCX (which contain strong and weak cation exchange groups) and Oasis MAX and WAX (which contain strong and weak anion exchange groups), are also attracting attention [100]. In some cases, proper adjustment of pH might be important to avoid deprotonation and protonation of acidic and basic compounds, respectively. Oasis MCX and MAX have been successfully used to extract a wide range of pharmaceuticals from liquid and solid environmental matrices [101,102]. Boles and Wells [103] developed a new protocol for the extraction of amphetamine and methamphetamine from wastewater influents and effluents using Oasis WCX cartridges. Another report described the efficiency of using Oasis WAX to extract several PPCPs from surface water samples [104]. Several studies have demonstrated the modification of these traditional cartridges for optimum recoveries. For instance, an additional sorbent was applied on top of a polymeric HLB column to improve the SPE of 15 PPCPs in wastewater samples [105]. Salas et al. [106] utilized both anionic and cationic exchange sorbents within a single cartridge for the simultaneous extraction of basic and acidic pharmaceuticals. In general, researchers need to consider the cartridge format and the sample volume passing through it. Other parameters, such as sorbent weight (mg), capacity (mL), and pore size (μm), can affect the efficiency of the columns, as they play a crucial role in establishing the surface area upon which the analytes interact [107].

The main advantage of SPE is the ability to use small sample volumes, which helps to reduce matrix effects and enhance the chromatographic detection. Sample pretreatment is another aspect that needs to be considered before performing SPE. For example, adding preservatives to water samples can prevent PPCP degradation, while ensuring sufficient filtration can reduce interference and improve recovery [108]. Several studies have reported the advantages and limitations of online SPE compared to classical SPE or offline SPE [109]. Online SPE can provide highly automated and reliable extraction efficiency, which saves solvents and time, uses smaller volumes, and reduces contamination [107,110]. However, online SPE might not be suitable for various types and conditions of sample matrices due to the requirements for adjustments to sample pH, valve switching times, and injection volumes [111].

Recent developments in sorbents have become important in the SPE process. In particular, the integration of green sample preparation (GSP) strategies with various sorbents is now being increasingly prioritized to develop high-performance and environmentally friendly SPE methods. In this context, MXene, a two-dimensional material composed of transition metal carbides with chemically stable and highly active functional groups, has been successfully employed as a green sorbent in micro-SPE (µ-SPE) for the detection of triclosan, triclocarban, 2-phenylphenol, bisphenol A, and 4-tert-octylphenol in water and wastewater sources [112]. The incorporation of metal-organic frameworks (MOFs) as green sorbent materials in dispersive µ-SPE has been applied successfully to extract PCPs from wastewater samples [113]. Molecularly imprinted polymers (MIPs), which have the advantages of improved mechanical properties, chemical stability, cost-effectiveness, and high selectivity, also meet the principles of GSP as green SPE sorbents [114]. MIP-μ-SPE has been applied successfully for the determination of hexestrol, nonylphenol, and bisphenol A in environmental water samples [115]. Carbon-based nanomaterials have also been developed as green SPE sorbents to extract polycyclic musks [116], quinolones [117], metronidazole, sulfamethoxazole, diclofenac, naproxen [118], parabens, fluoroquinolones [119], and bisphenol A [120] from different environmental matrices due to the advantages of lower toxicity, high surface area, and varied structural morphology [114]. These sorbents have been used to improve the sensitivity and efficiency of extraction techniques. These advanced SPE sorbents have been successfully applied for the extraction of several analytes from complex environmental matrices and have made a significant contribution to GSP.

### 3.2. Microwave-Assisted Extraction (MAE)

Microwave energy was first used in sample preparation in the 1970s [121]. Since then, microwave-based sample extraction has attracted growing interest and is now widely used in sample preparation. MAE is one of the green solvent extraction techniques that reduces extraction time while avoiding the extra release of solvents into the environment [122]. The simple and effective extraction achieved with MAE has largely overcome the limitations of conventional techniques, such as Soxhlet or liquid–liquid extraction (LLE) [123]. The basic principle of the MAE extraction process is that microwaves have a direct influence on molecules through ionic conduction and dipole rotation [124]. Given the limited number of parameters in MAE, including the nature of solvents, time, matrix moisture, open or closed vessels, and temperature, the optimization of an MAE protocol is rather simple. MAE has been applied to complex environmental matrices [125] and is viewed as an excellent alternative green extraction method for the determination of emerging PPCPs in different environmental water [126], soil [127,128], sediment, and sewage sludge [129,130] samples. Another study proposed a rapid and simple one-step microwave-assisted headspace SPME procedure for the extraction of different personal care products [131]. Novel MAE methods have recently been developed for the simultaneous extraction of PPCPs from different environmental matrices. For example, the MAE conditions were optimized for the determination of antibiotics using an orthogonal design [132]. MAE, combined with hollow fiber-liquid/solid-phase microextraction (MAE-HF-L/SME), has recently been proposed as a suitable tool for the extraction of 54 PPCPs [133]. This combination enhances the diffusion rate of the target compounds, thereby reducing extraction times while providing low matrix interference and a high enrichment factor. Combinations of MAE with other extraction techniques have been proposed for PPCP determination in several studies [130,134]. The combination of MAE and online SPE has been applied for the simultaneous determination of parabens, preservatives, UV filters, and plasticizers in sediment, soil, and sludge [135,136]. When compared with matrix solid-phase dispersion, MAE was proposed to extract parabens from soil samples with equivalently good recoveries and sensitivity [127]. In summary, the main advantages of MAE include reduced extraction time, minimized solvent usage, capacity for simultaneous extraction of multiple samples, enhanced recovery, and environmental friendliness, making MAE a green extraction technique [137,138]. However, MAE is limited to the extraction of a narrow range of compounds and thermally stable substances [30].

### 3.3. Dispersive Liquid–Liquid Microextraction (DLLME)

LLE is among the oldest extraction techniques in analytical chemistry. The introduction of microextraction techniques was developed to provide more efficient and miniaturized sample preparation methods [139,140,141,142]. Rezaee et al. [143] developed the first method for adding dispersive solvents to LLME (DLLME) as a simple and fast method for extracting and preconcentrating organic compounds from water samples. The major advantages of DLLME over conventional methods include its low cost, lower solvent consumption, and greater enrichment factor [143]. In addition, several advantages of different modes of DLLME have been reported. These include lower toxicity in low-density solvent-DLLME (LDS-DLLME) and SFO-DLLME, the ability to use green extractants in ionic liquid-DLLME (IL-DLLME) and deep eutectic solvent (DES-DLLME), and faster separation in magnetic nanoparticle-DLLME. However, DLLME still has some limitations, including the difficulty of separating the extraction phase in LDS-DLLME and reverse phase-DLLME, challenges in controlling temperature in SFO-DLLME, the high cost associated with IL-DLLME, and the high consumption of the organic phase in normal DLLME [144]. Nevertheless, DLLME contributes to green analytical chemistry, as it uses greener reagents (e.g., ethanol and ionic liquids) at reduced volumes [145]. To overcome some of these limitations, DLLME with solidification of floating organic droplets (DLLME-SFO) has been proposed for the extraction of PPCPs to avoid the use of environmentally unfriendly chlorinated solvents [84,146]. Similarly, Rozaini et al. [147] developed a simple, rapid, and green method that uses ultrasound-assisted salt-induced liquid–liquid microextraction (UA-SI-LLME) for the determination of triclosan, triclocarban, and methyl-triclosan in wastewater. In this technique, two solvents are typically used: a dispersant, which is highly miscible in both the aqueous sample and the organic extraction solvent, and an extractor. The process involves the formation of a cloudy solution after the rapid injection of the extraction solvent into the sample solution. The analyte is transferred between the aqueous and organic phases, and the mixture is centrifuged to separate the two phases, which are then collected for further analysis [148].

However, DLLME is limited by the number of extractor solvents that can be used, as they must be water immiscible, compatible with analytical instruments, and have the ability to extract analyte of interest. Accordingly, some modifications, such as automatization, safer and lower density solvents, and the use of ultrasound and vortex-assisted DLLME, have been proposed to optimize DLLME efficiency [77,78,149]. Application of DLLME for analysis of various PPCPs, such as antidepressants [150], NSAIDs [151], fragrances [152], and UV filters [153] in environmental samples, have been reported extensively.

### 3.4. Other Extraction Methods

Other extraction methods, including ultrasound-assisted extraction (UAE), supercritical fluid extraction (SFE), and pressure liquid extraction (PLE), have been widely reported as effective substitutions for conventional methods. UAE is regarded as a cheap and effective method capable of extracting thermally unstable analytes. The principle of UAE is based on the changes in operation kinetics, viscosity, cavitation, and temperature in response to the imposition of an ultrasonic field [154]. UAE methods have been developed for simultaneous extraction of many pharmaceuticals from freshwater sediment samples [155], sewage sludge [98], and soil [156]. Comparison of the UAE, QuEChERS, and selective PLE (SPLE) methods used for extraction of PPCPs in sludge [156] has revealed SPLE as the most suitable extraction method due to its high recoveries, lower detection and quantification limits, automated sample treatments, and simultaneous sample extractions. A developed method for the extraction of 66 PPCPs in WWTP confirmed PLE as an effective method [157]. Nevertheless, PLE has shortcomings in terms of lower extraction selectivity for some samples containing other compounds that might interfere with the analyte of interest [154]. The interference can be avoided by incorporating SPE with different sorbents for analyte preconcentration and purification [158,159]. Other studies have shown that SFE is fast and has a lower solvent consumption and less matrix interference [160]. However, many parameters still require optimization, particularly analyte collection [161]. Herrero et al. [162] reviewed the application of SFE in different areas, including the natural products, food science and pharmaceuticals, and environmental science fields. Others have described its fundamentals, advantages, disadvantages, and applications in pharmaceutical science [163].

The effectiveness of different clean-up methods after the extraction procedure has been evaluated in several studies [164]. Sample clean-up is frequently required to reduce interference effects and minimize detection limits. Two common approaches used for sample cleaning and preconcentration are gel permeation chromatography and SPE [165,166]. Post-extraction clean-up of PPCPs in sewage sludge has been performed to overcome the large interference and remove the bulk materials that are co-extracted with the analytes [18]. Finally, the selection of sample preparation and extraction techniques mainly depends on the specific objective of the study, with the aim of achieving the optimum conditions that provide accurate, precise, sensitive, and selective analytical results.

## 4. Instrumental Analysis

Even with an efficient and complete sample treatment, the instrumental techniques chosen for sample separation should be carefully selected to insure the optimum determination of the targeted analytes. Table 3 and Table 4 show the most commonly used methods for PPCP detection and quantification in environmental samples. Liquid chromatography (LC) is the most common analytical method, followed by gas chromatography (GC) coupled with mass spectrometry (MS or tandem MS^2^). Separation by capillary electrophoresis (CE) has the advantage of better selectivity over LC. Liquid and solid chromatography can be used to detect ECs based on the volatility, polarization, and thermal stability of the target compounds [167].

### 4.1. LC-MS and LC-MS^2^ Techniques

Instrumental analysis for PPCPs in several complex environmental matrices basically depends on chromatographic separation coupled to MS. MS is the preferred detection option and is now the most popular detection technique, as it enables direct analysis of a large spectrum of compounds with no prior derivatization process required. Recently, LC-MS has gained considerable attention because of the simplicity, sensitivity, and robustness of utilization allowed by different ionization techniques, such as electrospray ionization (ESI) and atmospheric pressure chemical ionization (APCI) [168]. ESI is the most commonly used soft ionization technique, as it generates ions with little or no fragmentation [169,170]. Nonetheless, the ionization efficiency of nonpolar compounds by ESI is limited, and APCI is then recommended [171]. Weak acids, such as formic acid (approximately pH 3), and weak bases, like ammonium acetate (approximately pH 8), are frequently used as mobile phase modifiers for +ESI and −ESI, respectively. Given the prevalence of basic functional groups, such as amines (pKa > 3), in numerous PPCPs, these compounds tend to ionize positively and are effectively analyzed using +ESI rather than −ESI [172]. A novel rapid and green method developed using APCI-MS/MS for the quantification of diclofenac in wastewater and wastewater sludge has been effectively performed in place of the conventional slow and matrix-sensitive ESI-MS/MS [172]. Other work has confirmed that ESI is less robust and more sensitive to matrix effects than APCI for the detection of sulfonamide and trimethoprim in manure [173]. However, certain classes of compounds, including some steroids and nonpolar compounds, are difficult to ionize using either ESI or APCI because of their lower ionization efficiencies [174].

Adduct formation during ESI can increase the background noise, leading to signal suppression or enhancement of the targeted analyte [175,176]. In addition, the high charge affinity of some compounds can suppress the ionization of the target analyte molecule. For these reasons, several studies have reported the importance of chemical derivatization or using different ion sources to increase the LC-MS sensitivity of various analytes [174,177,178]. Unfortunately, APCI has been found to effectively ionize only a limited number of analytes when used with MS or MS^2^, meaning that the majority of analytes need to be analyzed using ESI-MS^2^. Consequently, addressing the matrix effect is vital when validating newly developed analytical methods for the analysis of PPCPs in environmental samples, since the majority of LC-MS and LCMS^2^ applications must use the ESI technique.

Novel inceptions of the MS analyzer (e.g., single or triple quadrupole [QqQ], ion trap, orbitrap, and time of flight [TOF]) have also been incorporated to broaden the range of samples that can be detected and quantified (Table 3 and Table 4). Within mass modalities, MS^2^ has functioned efficiently for the targeted analysis of PPCPs in different environmental samples. Lower limits of detection (LODs) can be obtained using LC-MS^2^ working in selective reaction monitoring mode (SRM) than are possible using GC-MS. However, the versatility associated with less complicated sample analysis make LC-MS^2^ the most suitable application for environmental analysis of pharmaceutical residues and their metabolites (Table 3 and Table 4) [179]. Nevertheless, interest has been growing in utilizing other tandem combinations, such as Q-TOF, sparked recently by advancements in the dynamic range and sensitivity of TOF. In addition, TOF analyzers provide high-resolution capabilities, ensuring highly selective techniques with a lower probability of false-positive results. In contrast to QqQ, TOF enables the qualitative analysis of unknown compounds [172]. Combined targeted and nontargeted analyses of various PPCPs in drinking water systems have been performed effectively using QqQ and qTOF, respectively [180]. Despite the high sensitivity of QqQ and the possibility of confirmation on its own, LC-TOF-MS has been successfully combined and applied for the confirmation of several target pharmaceuticals that are not detected using QqQ. The main disadvantage of using both analyses is the high cost, which can be avoided by using TOF for analyzing analytes present in sufficiently high concentrations or reserving their use for analytes for which extra confirmation is needed [181].

The integration of liquid chromatography (LC) with high-resolution mass spectrometry (HRMS), which features high mass accuracy, is a highly effective technique for analyzing a mixture of known and unknown contaminants in environmental samples. The use of hybrid mass spectrometers, such as quadrupole/time-of-flight (qTOF) and linear ion trap/orbitrap (LTQ Orbitrap) instruments, has demonstrated exceptional performance in detecting and identifying low-molecular-weight compounds. This capability is largely attributed to the capability for high-resolution and accurate mass measurement of both precursor and product ions [182]. Targeted screening and identification of PPCPs in WWTP have been performed effectively using LC-Orbitrap HRMS [183]. The use of Q-Orbitrap is another effective combination for HRMS, as it gives the advantages of a wide dynamic range, high sensitivity, and improved selectivity. Moreover, Q-Orbitrap instruments, such as Q-Exactive Orbitrap™, have the important feature of rapid polarity switching (<1 s) between positive and negative ionization modes in ESI [184]. The orbitrap-quadrupole combination also has the advantage of possible preselection of several precursor ions [185], thereby offering efficient analysis of complex mixtures, like PPCPs, in environmental matrices [184].

The mobile phase is normally modified to improve compound separation and the sensitivity of MS detection. LC-MS^2^ also allows separation and detection of coeluting analytes with similar molecular masses, as they produce different product ions. Complex samples require efficient separation of the analytes to reduce the suppression effect from other compounds and improve the signal. Thus, using LC-MS^2^ techniques can reduce matrix effects and enhance detectability. Matrix effects and signal suppression can be reduced by several techniques, such as using an anion exchange cartridge with HLB in wastewater samples for the determination of sulfonamide, fluoroquinolone, and trimethoprim [186]. Another study of the effects of mobile phase composition and signal suppression on the analysis of a broad range of acidic/neutral PPCPs in surface water SPE/LC/ESI/MS-MS [187] showed that the use of an internal standard, selective extraction, and dilution of sample extracts could enhance sample clean-up and eliminate the matrix effect, particularly when ESI was employed [188]. In summary, a number of methods can be used to address the issues that can hinder quantification.

The utilization of the rapid and efficient capabilities of LC-MS techniques facilitates the development of green and eco-friendly methods for PPCP detection in environmental matrices. Several LC–MS/MS approaches, including reducing the internal diameter and particle size of chromatographic columns to decrease eluent consumption, have been employed to lessen the environmental impact [189]. Toxic reagents and conventional mobile phases, such as acetonitrile and methanol, have also been replaced by smaller amounts of safer and environmentally friendly reagents, such as water and ethanol [190,191]. The use of high-speed LC, eco-friendly hydrophilic interaction chromatography (HILIC) [192], and direct liquid chromatography [193] could also be beneficial as green approaches in LC separation. Consequently, the greenness of analytical procedures must be assessed using several green metric tools, such as the analytical Eco-scale, to select the most eco-friendly new method [75].

### 4.2. GC-MS and GC-MS^2^ Techniques

GC-MS and GC-MS^2^ are capable of detecting nonpolar and volatile PPCPs down to levels below the ng/g scale. The majority of PPCPs are polar, nonvolatile, and thermally unstable, making them unsuitable for GC analysis. Thus, many of these compounds first require derivatization by alkylation, acylation, or silylation to overcome this limitation [194,195,196,197]. The derivatization reaction used in GC is chosen to enhance the selectivity, sensitivity, and peak separation of the analytes [198]. However, the derivatization step might also increase the complexity of the sample, require longer analysis time, and incur more errors. GC has been utilized for the analysis of phenolic compounds [199,200], musk fragrances [201], and UV filters [202] in environmental matrices. In GC, electron impact (EI) ionization coupled with MS can be applied to analyze many compounds with no apparent matrix effect. However, sample preparation for GC analysis usually demands optimum sample extraction, clean-up, and preconcentration processes. For instance, PLE has been applied in combination with solid-phase clean-up of manure and soil samples prior to derivatization and analysis of steroid hormones using GC-MS^2^ [203]. A method using SPME, derivatization, and GC-MS was deemed effective for the determination of fragrances, UV filters, antiseptics, estrogens, and anti-inflammatory drugs in water samples [204].

Given the lower detection limits for PPCPs in water samples using LC-TOF-MS, library identification of additional unknown peaks in GC-MS, such as the National Software Reference Library (NIST), would be advantageous [205]. Tsizin et al. [206] reported that a broader range of small compounds can be identified, even at the isomer structural level, using cold EI in GC-MS rather than ESI-LC-MS, due to the compatibility of EI with library-based sample identification and the ability to analyze relatively nonpolar compounds. In general, good sensitivity and analytical speed would support the wide application of GC-MS [207]. Comprehensive two-dimensional gas chromatography (GC × GC) has become a powerful emerging technique for the separation and identification of analytes in complex matrices. Several studies have confirmed that this technique increases the chromatographic resolution and improves analyte detectability. For instance, the simultaneous determination of various pharmaceuticals in river water was performed successfully using GC × GC coupled with TOF-MS [208]. Benzothiazoles, benzotriazoles, and benzosulfonamides have been identified in aqueous matrices using GC × GC-TOF-MS with enhanced chromatographic separation and improved accuracy compared with 1D-GC and LC techniques [208,209]. At present, GC × GC-TOF-MS has been proven to resolve up to an order of magnitude more compounds from complex matrices when compared to conventional methods [210]. Consequently, an efficient detector is required that can handle the large packet of data generated by GC × GC. Furthermore, coeluted compounds can be separated by MS with the addition of automatic spectral deconvolution [210]. The present time-consuming determination of the identities of peaks and isolated compounds of interest could be overcome by coupling GC × GC with fast scanning TOF, which should provide sufficient capability for the identification of large sets of compounds [211]. The growing interest in GC × GC in recent years indicates that this technique is becoming more important in environmental laboratories, although it is still not widely adopted as a routine analysis method in most cases.

Overall, modern analytical techniques have made possible the identification and quantification of low levels of PPCPs in complex environmental samples. Integrating the principles of green chemistry into these techniques can aid in reducing their environmental impact. In this regard, various strategies have been reported for applying green analytical chemistry in GC, including direct analysis methods, reducing sample preparation and solvent consumption prior to GC analysis, minimizing gas emissions, shortening run times, and using shorter columns packed with smaller particles [212,213]. In addition, hydrogen has been proposed as a safe and green gas to replace helium, as it can increase the linear flow rate without contributing to environmental pollution [214]. Considering these approaches and focusing on the essential aspects of shifting toward green GC will aid in reducing the environmental impacts associated with analytical chemistry.

### 4.3. Other Techniques

Many other instrumental techniques, including capillary electrophoresis (CE), can be used for the determination of PPCPs in numerous environmental samples. The CE method is simple, fast, and effective and shows a high resolution for a wide range of compounds [215]. As an alternative to LC-MS^2^, CE can offer better selectivity for the identification of PPCPs in environmental matrices by optimization of the additives, buffer concentrations, and pH tuning [216]. A number of CE methods have been developed to identify PPCPs in feeds [217], groundwater [218], surface water [219], and wastewater [216]. CE systems coupled to different detectors for UV detection [216], UV-diode array detection (DAD) [220,221], and MS detection [222] have been used effectively for several pharmaceutical compounds in environmental samples. However, the lower sensitivity of CE compared to HPLC suggests that CE should not be the first choice for the analysis of environmental sample components [167]. Nevertheless, CE is a useful technique for confirming HPLC results when required.

Ion mobility spectrometry (IMS) is a rapid separation technique that separates ions in a gas phase based on their size, shape, and charge [223]. IMS has received growing interest over recent years for the determination of various environmental micropollutants. Its application has increased the peak capacity of LCMS^2^ by adding another separation dimension [224]. The combination of IMS with HRMS can reduce background noise and matrix interference and remove coeluted compounds [225]. Another attractive strength of IMS-MS in the analysis of environmental samples is its ability to separate and identify isomers [226]. However, the extended length of mobility devices and longer analysis times hinder the integration of IMS techniques with liquid or gas chromatographic systems; thus, its application in environmental analysis is limited. Alternative detectors to MS detectors have shown a certain degree of usefulness in analyzing specific PPCPs. For example, flame ionization detectors (FIDs) and electron capture detectors have been coupled to GC systems for the extraction of pollutants from environmental samples [227,228]. Many studies have confirmed the efficiency of using HPLC-DAD [229,230,231] and fluorescence detectors [232] for the simultaneous determination of PPCPs in environmental samples. Another study demonstrated the ability of fluorescence spectroscopy to assess high concentrations of pharmaceutical contaminants in water samples [233]. However, these alternative techniques have several disadvantages, including poor sensitivity and a lack of selectivity on occasion. Thus, the use of more sensitive and selective techniques, such as choosing a different MS ionizer (e.g., EI and ESI) and MS analyzer (e.g., orbitrap, quadrupole, QqQ, ion trap, and TOF), has become increasingly important.

## 5. Summary of Analytical Challenges

One crucial challenge is identifying suitable techniques that need to be considered before exploring method development. Consideration is particularly needed to determine whether the analyte can be quantitatively analyzed using GC, or if it can be derivatized for more amenable analysis. Conversely, the use of LC is imperative for analyzing high-molecular-weight analytes, samples that require extensive derivatization, or compounds that are nonvolatile or thermally labile. In a complex sample matrix, three major problems hinder the accuracy of analytical methods: (1) the complex mixture of PPCPs, (2) the low concentration of analytes, and (3) the matrix effects. These challenges must be overcome, and this is usually accomplished by optimizing the sample preparation, sample clean-up, and sample pretreatment, and by the use of state-of-the-art techniques. All steps in sample preparation and instrumental analysis should be taken into consideration to obtain more reliable and reproducible methods for the determination of target analytes.

Currently, the challenge of tracing low levels of PPCPs in complex environmental samples has shifted from attaining a highly sensitive detection method to using greener methods. A green analytical approach can be achieved using suitable solvents, less solvent, shorter analysis times, and a minimum number of steps during sample preparation and detection. However, despite the optimized method already performed with high extraction efficiency and the techniques available, the sensitive, accurate, and rapid determination of very low concentrations of PPCPs in complex environmental matrices continues to be a challenge. Moreover, sample contamination and loss should also be considered for optimum analytical methods. This can be achieved by coupling two or more techniques, such as on-line SFE with supercritical fluid chromatography-MS (SFE-SFC-MS) [234]. In this review, different sample preparations were summarized, including several advantages; however, SPE is the predominant method, especially for interference removal.

The final sample preparation step is the clean-up, which is added to enhance reproducibility and accuracy by eliminating any impurities and matrix effects occurring in the final extract. Matrix effects can suppress, mask, or augment analyte signal measurement. This can be shown chromatographically through coelution, ionization processes, and detection, and results in variable and unreliable data. As mentioned above, the matrix effect can be reduced by the use of suitable techniques for particular analytes [186], optimizing the conditions for sample preparations and analytical methods [187] and the use of stable isotope internal standards [188]. The use of isotope-labeled standards (ILS) is one of the most effective strategies for correcting the matrix effect, while also correcting instrument drift and analyte loss during the preparation of environmental samples [235,236]. For example, an SPE-LC-ESI-MS/MS method developed to determine 27 PPCPs in environmental water samples used 27 ILSs to correct the quantitation in the presence of various interferences [237]. The quantitative analysis of 24 compounds in water samples was achieved using 13 ILSs with recoveries of more than 70% and quantification limits below 10 ng/L [83]. However, identifying suitable internal standards for each analyte in a multi-component study can be challenging. For this reason, matrix-matched calibration with one internal standard, among other suitable calibration approaches to compensate for matrix effects, is a practical alternative option for the multi-component analysis of environmental samples [238]. Matrix-matched calibration curves have been used to counterbalance the matrix-induced enhancement effect of selected PPCPs in water samples when using ion trap tandem mass spectrometry [239]. The recommended procedure for matrix-matched calibration is to spike the matrix with varying concentrations of target analytes and then subject them to the complete sample extraction process, as this can effectively address matrix effects and sample preparation-related losses in environmental analysis [240]. This approach has been followed and compared with ILSs for the determination of antibiotics and antiretroviral drugs in surface water [241]. Comparison of the quantification to internal standards had a margin of error of ±20% [241]. Background subtraction, isotope-labeled internal standards, and surrogate matrices can all be used as calibration approaches when a blank matrix is not available [240]. These steps can reduce the coelution of compounds originating from the matrix in complex environmental samples, leading to significant decreases in signal enhancement or suppression. Nevertheless, the developed method needs to be optimized for effective determination and separation of the coeluted targeted PPCPs, as well as their metabolites and transformation products. Furthermore, a large spectrum of complex samples can be analyzed by performing an efficient technique with less signal suppression, such as multiple reaction monitoring (MRM), spectral filtering, and deconvolution of coeluted signals.

In summary, sample preparation needs to be selected based on the nature of the targeted analytes, the type of matrix, and the concentration of the analyte in the matrix (from trace to ultra-trace concentrations). For the analytical method, when including GC, LC, and their detectors, the major advantages and disadvantages have to be evaluated, in addition to the above-mentioned factors, in terms of analysis time, ease of automation, and cost-effectiveness. Overall, the current analytical challenges are focused on optimizing and validating new strategies and procedures to meet the requirements of highly sensitive, selective, and green methods.

## 6. Conclusion and Future Prospects

The occurrence of PPCPs in different environmental sources and the environmental risks they pose have been summarized, and a comprehensive review has been provided regarding the analytical methods for the determination of PPCPs as emerging contaminants. PPCPs have become a crucial part of modern life and have consequently shown rapid increases in their rates of consumption over the last century. Accordingly, the discharge of PPCPs and their transformed metabolites into the environment has increased significantly. The risks posed by PPCP contaminants to human health have been confirmed in several studies, even when these materials are present at only trace levels (ng/L–μg/L). Obtaining a better understanding of the impact of PPCPs requires the ability to identify and quantify them within different environmental matrices. Therefore, combinations of analytical methods, including SPE, LC, and MS^2^, have emerged as methods of choice for environmental sample extraction and separation and detection of target analytes. SPE-based LC-MS/MS provides a robust, sensitive, selective, and broadly applicable technique for reliable assessments of environmental PPCP contaminants.

Ongoing analytical challenges include the difficulty in measuring trace levels of contaminants (ng/L), the complexity of environmental samples, and the matrix effects. These difficulties can be minimized by optimizing sample clean-up methods and using IS, which offers better recovery and fewer interference effects. Ongoing advancements in various sorbents continue to represent a promising approach for the extraction of PPCPs. Currently, hybrid instruments, such as QqTOF and LTQ Orbitrap, are expected to play a significant role in addressing knowledge gaps by providing accurate mass measurements of trace analytes and acquiring essential qualitative information through full-scan mass spectra. However, the screening of PPCPs and their transformed metabolites in environmental samples still needs advanced tools, and much work remains to develop more accurate combined databases. Automated batch processing protocols using advanced software solutions are also required to facilitate the nontargeted analysis of complex environmental matrices within a reasonable timeframe. The significant impact of HRMS in environmental analysis will clearly grow in the coming years as the technique improves in its reliability and robustness, particularly with respect to nontargeted analysis. In addition, the greening of analytical methods, while retaining chromatographic performance, is gaining great interest. When comparing and evaluating different parameters and analytical processes, the use of green analytical metrics is highly recommended to meet green chemistry requirements. Finally, substantial effort is still needed to ensure the consistency in the analysis of PPCP micropollutants present in environmental matrices and to develop innovative high-performance detection and analysis methods.

## Figures and Tables

**Table 1:** Chemical structures and physicochemical properties of selected PPCPs.

Chemical structure	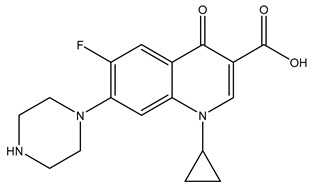	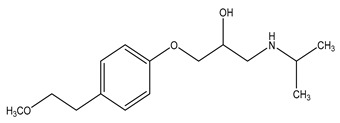	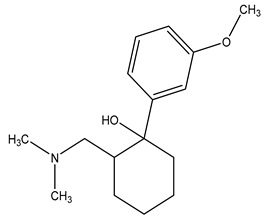
Name	Ciprofloxacin	Metoprolol	Tramadol
Formula	C_17_H_18_FN_3_O_3_	C_15_H_25_NO_3_	C_16_H_25_NO_2_
CAS	85721-33-1	51384-51-1	123154-38-1
Molecular Weight	331.34 g/mol	267.36 g/mol	263.37 g/mol
Water solubility at 25 °C (mg/mL) ^a^	36	>1000	0.036
pKa ^a^	6.09; 8.74	9.7	9.23
Log Kow ^a^	0.28	1.88	3.01
Log Koc ^a^	4.78	1.79	2.79
Chemical structure	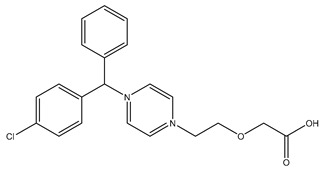	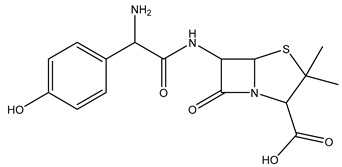	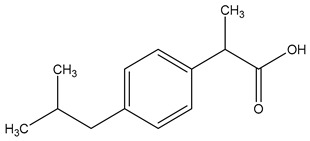
Name	Cetirizine	Amoxicillin	Ibuprofen
Formula	C_21_H_25_ClN_2_O_3_	C_16_H_19_N_3_O_5_S	C_13_H_18_O_2_
CAS	83881-51-0	26787-78-0	15687-27-1
Molecular Weight	388.9 g/mol	365.4 g/mol	206.28 g/mol
Water solubility at 25 °C (mg/mL) ^a^	0.101	0.011	0.021
pKa ^a^	1.52; 2.92; 8.27	2.6	4.91
Log Kow ^a^	1.70	0.87	3.97
Log Koc ^a^	2.30	2	Log 3400
Chemical structure	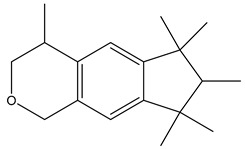	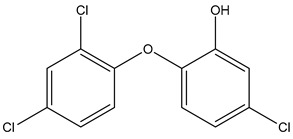	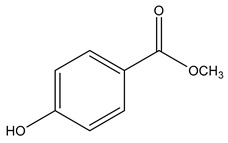
Name	Galaxolide	Triclosan	Methylparabens
Formula	C_18_H_26_O	C_12_H_7_Cl_3_O_2_	C_8_H_8_O_3_
CAS	1222-05-5	3380-34-5	99-76-3
Molecular Weight	258.4 g/mol	289.5 g/mol	152.15 g/mol
Water solubility at 25 °C (mg/mL) ^a^	0.00175	0.01	2.5
pKa ^a^	−6.9	7.9	8.5
Log Kow ^a^	5.90	4.76	1.96
Log Koc ^a^	Log 2.0 × 10^4^	3.54	Log 87

Ka = Acid dissociation constant; Kow = Octanol-water coefficient; Koc = Organic carbon partition coefficient. ^a^ https://pubchem.ncbi.nlm.nih.gov/ (accessed on 1 May 2024).

**Table 2:** Occurrence and concentrations of the most commonly detected PPCPs in different matrices from different countries (2010–present).

PPCPs	Classification	Occurrence	Concentration	Country	Ref.
**Pharmaceuticals**					
Ofloxacin and ciprofloxacin	Antibiotics	WWTP influent	1000–2200 ng/L	Italy	[31]
Ciprofloxacin, clarithromycin and sulfamethoxazole	Antibiotics	Wastewater influent	570–1200 ng/L	Canada	[32]
Ciprofloxacin	Antibiotic	Biosolids	6500 ng/g	Canada	[32]
Amoxicillin	Antibiotics	STP	172 ng/L	India	[33]
Ketoprofen, diclofenac, and indomethacin	Analgesic/NSAIDs	Sludge	4.4–77 ng/g	Japan	[34]
Sulfapyridine, sulfadiazine, sulfamethoxazole, sulfamethazine, and three sulfonamide acetylated metabolites	Antibiotics	WWTP (effluent and influent)	3–124 ng/L	China	[35]
Metoprolol, atenolol, betaxolol, Sotalol, Pindolol, and Nadolol	β-blocker	Hospital wastewater	3.4–6700 ng/L	Italy	[31]
Atenolol and carbamazepine	β-blocker, antibiotics	Soil	0.1–64.6 ng/g	Spain	[36]
Tramadol, ofloxacin, gemfibrozil, atenolol, caffeine, and cetirizine	Analgesic, antibiotics, lipid regulator, β-blocker, stimulant and antihistamine	Irrigation water	1100–4400 ng/L	Spain	[37]
Tramadol, cetirizine, and clarithromycin	Analgesic, antihistamine and antibiotics	Soil	12.7–14.6 ng/g	Spain	[37]
Estrone, 17b-estradiol, estriol, 2-hydroxyestrone, 16α-hydroxyestrone, 4-hydroxyestrone, 2-hydroxyestradiol, 4- hydroxyestradiol, 17-epiestriol, 16keto-estradiol, and 16-epiestriol	Steroidal hormones	WWTP (effluent and influent)	n.d.–62.9 ng/L	China	[38]
		River water	n.d.–51.7 ng/L		
Atenolol, ciprofloxacin, sulfamethoxazole, ranitidine	β-blocker, Antibiotics and antihistamine	WWTP (effluent and influent)	34–3585 ng/L	Saudi Arabia	[39]
Metformin and acetaminophen	Antihyperglycemic and analgesic	Sea water	<LOQ–>3000 ng/L	Saudi Arabia	[40]
**Personal care products (PCPs)**					
3-Benzophenone galaxolide and tonalide	UVA/UVB filters, Synthetic fragrances	Irrigation water	1200–18,900 ng/L	Spain	[37]
Galaxolide	Fragrances	Soil	2.8 ng/g	Spain	[37]
Galaxolide and tonalide	Fragrances	Sediment	2.39–52.5 ng/g	Brazil	[41]
Oxybenzone, triclosan, and triclocarban	UV filters and disinfectants	Wastewater (effluent and influent) and sludge	n.d.–8880 ng/L	India	[42]
Triclosan and parabens	Disinfectants and preservatives	Surface water and sediment	n.d.–5160 ng/L	India	[43]
Triclocarban and triclosan	Disinfectants	Biosolids	12,640–36,060 ng/g	USA	[44]
Triclosan and triclocarban	Disinfectants	Biosolids	2900–6800 ng/g	Canada	[32]
Benzotriazole and methyl benzotriazol	UV filters	Groundwater	267–1980 ng/L	Spain	[45]
Triclosan (TCS) and triclocarban (TCC)	Disinfectants	Biosolids	760–22,588 ng/g	USA	[46]
Triclosan (TCS) and triclocarban (TCC)	Disinfectants	Soils	2.8–221 ng/g		
Diethyltoluamide	Insect repellent	Surface water	1.4–527 ng/L	Singapore	[47]
Tonalide, galaxolide	Fragrances	Groundwater	1–50 ng/L	France	[48,49]
Musk ambrette, musk ketone, and musk xylen			209–1304 ng/L		

n.d.: Not detected; NSAIDs: Nonsteroidal Anti-inflammatory Drugs.

**Table 3:** Analytical methods applied for the determination of the most commonly detected PPCPs in water samples (2010–present).

Analyte	Matrix	Extraction Method	Analytical Technique	Analytical Conditions	Analysis Time (min)	Analytical Parameters	Ref.
Ofloxacin and ciprofloxacin	WWTP influent	SPE Oasis HLB	LC-ESI-MS/MS	C18 column (125 mm× 2.0 mm, particle size 5 μm) MP: acetonitrile (A) and HPLC grade water with 0.1% formic acid (B) Flow rate= 0.2 mL/min	45 min	Recovery = 93–107% LOD = 1–3 ng/L	[31]
Ibuprofen, carbamazepine, and triclosan	WWTP effluent	SPE Oasis HLB	LC-ESI-MS/MS	C18 Column (100 mm× 2.1 mm, particle size 3 μm). MP: acetonitrile/methanol (A) and HPLC grade water with 0.1% formic acid (B), Flow rate = 0.25 mL/min	35 min	Recovery = >80% LOQ = 1–2 ng/L	[79]
Amoxicillin	STP	SPE Oasis HLB	HPLC-PDA	λ: 228 nm C18 Column (250 mm × 4.6 mm, 5 μm) MP: isocratic program of 0.45% Na_2_HPO_4_ in water (A) and Methanol (B) in the fixed ratio of 2:1 Flow rate = 1.5 mL/min	4.9 min	Recovery = 64–88% LOD = 1 ng/L LOQ =10 ng/L R^2^ = 0.998	[33]
Ampicillin, ciprofloxacin, gatifloxacin, sparfloxacin and cefuroxime	STP	SPE Oasis HLB	HPLC-PDA	λ: 215 and 280 nm C18 Column (250 mm × 4.6 mm, 5 μm) MP: 0.1% aqueous TFA (A) and acetonitrile (B) Flow rate = 1.0 mL/min	30 min	Recovery = 25–108% LOD = 10 ng/L LOQ =30 ng/L R^2^ = 0.9806–0.9957	[80]
Sulfapyridine, sulfadiazine, sulfamethoxazole, sulfamethazine, and three sulfonamide acetylated metabolites	WWTP (effluent and influent)	SPE Oasis HLB	LC-ESI-MS/MS	C18 column (100 mm × 2.1 mm, particle size 2.6 μm) MP: methanol (A) and distilled water (B) Flow rate = 0.3 mL/min	6 min	Recovery = 77.7–148.1% LOD = 0.01–0.23 ng/L LOQ = 0.03–0.78 ng/L R^2^ = 0.995–0.999	[35]
Sulfathiazole, sulfamethazine, sulfamethoxazole, oxytetracycline, and chlortetracycline	WWTP effluent	SPE Oasis HLB and MCX	LC-ESI-IT–TOF/MS	C18 column (75 mm× 3.0 mm, particle size 3.0 μm). MP: 0.3% formic acid and 0.1% ammonium acetate in water (A) and methanol and Acetonitrile (1:1) (B).	6 min	Recovery = 63–118% LOD = 9.8–25.8 ng/L R^2^ = 0.9964–0.9996	[81]
Estrone, 17β-Estradiol, estriol, 2-Hydroxyestrone, 16α-Hydroxyestrone, 4-Hydroxyestrone, 2-Hydroxyestradiol, 4- Hydroxyestradiol, 17-Epiestriol, 16keto-estradiol, and 16-Epiestriol	WWTP (effluent and influent) and river water	SPE Oasis HLB Derivatization	GC-MS	HP-5 MS capillary column (30 m × 0.25 mm × 0.25 μm) Flow rate = 0.85 mL/min Temperature program: start with 100 °C, rising at 10 °C/min to 200 °C, then 8 °C/min to 260 °C, then 3 °C/min to 310 °C, and finally held at 310 °C for 2 min	37 min	LOD =1.4–6.0 ng/L LOQ =4.8–19.8 ng/L R^2^ = 0.9838–0.9995	[38]
Atenolol, bisoprolol, ciprofloxacin, ofloxacin, sulfamethoxazole, clarithromycin, trimethoprim, ketoprofen, diphenhydramine HCl, ranitidine, and carbamazepine	WWTP (effluent and influent)	SPE Oasis HLB	LC-ESI-MS/MS	C18 column (30 mm × 2.1 mm, particle size 1.9 μm) MP: 0.1% formic acid in water (A) and ethanol (B) Flow rate = 0.5 mL/min	7.2 min	Recovery = 70.4–112.2% LOD = 0.06–16.0 ng/L LOQ = 0.20–52.8 ng/L R^2^ = 0.9921–0.9992	[39]
Sulfapyridine, sulfamethazine, sulfamethoxazole and sulfaphenazole	Surface water	Deep eutectic solvent (DES) and liquid–liquid microextraction	UHPLC-PDA	λ: 270 nm. C18 column (30 mm × 2.1 mm, particle size 1.7 μm) MP: 0.1% formic acid in water (A) and 90% acetonitrile in ethanol (B) Flow rate = 1 mL/min	3.5 min	Recovery = 78.2–105.2% LOD = 0.78–3.42 ng/L LOQ = 2.38–10.37 ng/L R^2^ = 0.9991–0.9997	[77]
Lincomycin, ibuprofen, paraxanthin, and naproxen	Lake	SPE Oasis HLB	LC-ESI-MS/MS	C18 column (150 mm × 2.1 mm, particle size 3.5 μm) MP: ammonium formate/formic acid buffer (A) and acetonitrile (B) Flow rate = 0.2 mL/min	50 min	Recovery = 78–118% LOD = 1.7–25 ng/L LOQ = 2.5–25 ng/L R^2^ ≥ 0.99	[82]
Acetaminophen, caffeine, carbamazepine, ibuprofen, ketoprofen, fenoprofen, naproxen, propyphenazone, clofibric acid, gemfibrozil, diclofenac, indomethacin, salicylic acid, crotamiton, and trimethoprim	Raw wastewater, surface water and groundwater samples	SPE Oasis HLB	LC-ESI-MS/MS	C18 column (150 mm × 2.1 mm, particle size 3.5 μm). MP: ammonium acetate in water (A) and ammonium acetate in methanol (B) Flow rate = 0.25 mL/min	18 min	Recovery = 66.5–102% LOD = 0.1–5 ng/L LOQ = 0.3–10 ng/L R^2^ ≥ 0.996	[83]
Estrone, 17 α-estradiol, estriol, progesterone, pquiline, diethylstilbestrol, and ethinylestradiol	Sea water, mineral water, and tap water	Air-agitated liquid–liquid microextraction method based on solidification of a floating organic droplet (AALLME-SFO)	LC-ESI-MS/MS	C18 column (50 mm × 2.1 mm, particle size 1.7 μm) MP: 0.1% formic acid in water (A) and ethanol (B) Flow rate = 0.4 mL/min Flow rate = 0.05 mL/min	6.7 min	Recovery = 90.14–99.60% LOD = 0.021–0.034 ng/L LOQ = 0.046–0.103 ng/L R^2^ = 0.9990–0.9998	[84]
Atrazine, bisphenol-A, caffeine, carbamazepine, gemfibrozil, ibuprofen, naproxen, simazine, sulfamethoxazole, and trimethoprim	Raw and treated water	SPE Oasis HLB	LC-MS/MS	N.R.	N.R.	Recovery = 70–128% LOD = 1–2.5 ng/L	[85]
Sulfadiazine, sulfapyridine, sulfathiazole, trimethoprim, sulfamerazine, sulfamethazine, sulfamethoxazole, naproxen, paracetamol, diclofenac acid, ibuprofen, ketoprofen, flumequine, ciprofloxacin, norfloxacin, ofloxacin, macrolides, clarithromycin, roxithromycin, erythromycin, tetracyclines, oxytetracycline, isochlortetracycline, tetracycline, cephalexin monohydrate, cefotaxime sodium, penicillin G, amphenicols, chloramphenicol, thiamphenicol, triclocarban, triclosan, carbamazepine, aminoglycoside, spectinomycin, antihyperlipidemic, gemfibrozil, diltiazem, diphenhydramine, and bisphenol A.	Lake	SPE Oasis HLB	LC-ESI-MS/MS	C18 column (50 mm × 2.1 mm, particle size 1.8 μm). MP: Water (A) and Acetonitril (B) fro −ESI and 0.1% formic acid in water (A) and acetonitrile (B) for +ESI. Flow rate = 0.25 mL/min	N.R.	Recovery = 70–125% LOD = 0.01–1.1 ng/L LOQ = 0.03–3.3 ng/L R^2^ = 0.9937–0.9999	[86]
Ibuprofen, diclofenac, fexofenadine, atorvastatin, irbesartan, triclosan, sulfasalazine, benzophenone 1, 2, and 4, methylparaben, diazepam, and oxazepam	WWTP (effluent and influent) and surface water	SPE Oasis HLB	LC-ESI-MS/MS	Separation of acidic analytes: C18 column (150 mm × 1.0 mm, particle size 1.7 μm). MP: (80:20) Water:Methanol conatin 1 mM Ammonium fluoride (A) and (5:95) Water:Methanol conatin 1 mM Ammonium fluoride (B) Flow rate = 0.04 mL/min Separation of basicanalytes: CHIRALPAK CBH HPLC column. MP: 1 mM ammonium acetate/methanol 85:15 (*v*/*v*) (Isocratic). Flow rate = 0.1 mL/min	N.R.	Recovery = 44.6–120% LOQ = 0.31–41.43 ng/L	[87]
Azithromycin, clarithromycin, metronidazole, ciprofloxacin, norfloxacin, vancomycin, and sulfamethoxazole	WWTP (effluent and influent) and surface water	SPE Oasis HLB	LC-coupled to hybrid MS/MS/Ion trap	C18 column (100 mm × 4.6 mm, particle size 2.6 μm). MP: 0.2% formic acid (A) and acetonitrile with 0.2% formic acid (B) Flow rate = 0.5 mL/min	35 min	LOD = 0.03–15 ng/L LOQ = 0.9–50 ng/L	[88]
Acetylsalicylic acid, naproxen, methyl and ethyl paraben, estrone, 17β-estradiol	Drinking water	SPE-DEX	LC-ESI-MS/MS	In −ESI: C18 column (50 mm × 2.1 mm, particle size 2.7 μm). MP: 10 mM N-methylmorpholine (A) and methanol (B). Flow rate = 0.5 mL/min In +ESI: Kinetex PFP HPLC column column (50 mm × 2.1 mm, particle size 2.6 μm). MP: 5 mM ammonium acetate (A) and methanol (B). Flow rate = 0.8 mL/min	5.5 min (−ESI) 6.5 min (+ESI)	Recovery = 32.9–130.1% LOD = 0.2–0.9 ng/L LOQ = 1.0–2.3 ng/L R^2^ = 0.9976–0.9996	[89]
Chloroform, tribromomethane, and dichloroacetonitrile	Drinking water	SPE-DEX	GC-ECD	DB1701 column	N.R.	LOD = 0.01–0.03 μg/L LOQ = 0.2 μg/L R^2^ = 0.9926–0.9992	[89]

MP: mobile phase; LOD: limit of detection; LOQ: limit of quantification; λ: wavelength; ESI: electrospray ionization; IT: ion trap; TOF: time of Flight; PDA: photo diode array; ECD: electron capture detector; N.R.: not reported.

**Table 4:** Analytical methods applied for the determination of the most commonly detected PPCPs in sludge, soil, sediment, and others (2010–present).

Analyte	Matrix	Extraction Method	Analytical Technique	Analytical Conditions	Analysis Time (min)	Analytical Parameters	Ref.
Ciprofloxacin, clarithromycin, indomethacin, ketoprofen, tramadol, diazepam, carbamazepine, ranitidine, and furosemide	Plant (root and stem/leaf)	QuEChERS	LC-ESI-MS/MS	C18 column (2.1 mm × 100 mm, particle size 1.8 μm) MP: 0.1% formic acid in ultrapure water (A) and acetonitrile (B) Flow rate = 0.3 mL/min	18 min	Recovery = 41.0–120.0% LOQ = 0.05–50 ng/g R^2^ = 0.9980–1.000	[90]
Tramadol, cetirizine, and clarithromycin	Soil	QuEChERS	LC-ESI- MS/MS	C18 column (2.1 mm × 100 mm, particle size 1.8 μm) MP: 0.1% formic acid in ultrapure water (A) and acetonitrile (B) Flow rate = 0.3 mL/min	18 min	Recovery = 70–120% LOQ < 0.5 ng/g R^2^ > 0.99	[37]
Acetaminophen, azithromycin, diclofenac, furosemide, norfloxacin, salbutamol, and sulfamerazine	Sludge	Ultrasonication and SPE Oasis HLB	Micro LC- MS/MS	C18 column (2.1 mm × 100 mm, particle size 1.7 μm)	N.R.	Recovery = 86.2–114.5% RSD = 2.9–29.8%	[91]
Ibuprofen, atenolol, carbamazepine, erythromycin, diclofenac, and diazepam	Sediment	Samples extracted with methanol and evaporated until dry, then diluted with methanol	LC-ESI- MS/MS	C18 column (150 mm × 4.6 mm, particle size 3 μm) MP: 5 mM ammonium acetate in water (A) and 5 mM ammonium acetate in methanol (B) Flow rate = 1 mL/min	N.R.	Recovery = 87–97% LOQ = 0.1 ng/g Reproducibility > 90%	[41]
Tonalide, galaxolide, and caffeine	Sediment	Samples extracted with methanol and evaporated until dry, then diluted with methanol	GC-MS/MS	DB-XLB column (60 m × 0.25 mm × 0.25 μm) Flow rate = 1 mL/min	N.R.		
Ketoprofen, diclofenac, and indomethacin	Sludge	SPE Oasis HLB	LC-ESI- MS/MS	C8 column (150 mm × 2.0 mm, particle size 3 μm) MP: aqueous NH_4_H_2_O_2_/CH_2_O_2_ buffer; 10 mM, pH 3.7 (A) and acetonitrile (B)	N.R.	Recovery = 52–137% (at pH-7) LOD = 0.16–0.5 ng/g LOQ = 0.49–1.53 ng/g	[34,92]
Estrogens, caffeine, triclosan, and ciprofloxacin	Sludge and soil	SPE Oasis HLB	HPLC-UV	λ: 200, 254 and 279 nm. C18 column (25 × 4.6 mm, 5 μm) MP: Acetonitrile (A) and Water (B) Flow rate = 0.8–1.0 mL/min	21 min	Recovery = 28–104% LOD = 0.30–6.42 ng/g	[93]
Carbamazepine, clarithromycin, carbamazepine-10,11-epoxide, clindamycin, diclofenac, diltiazem, diphenhydramine, erythromycin, fluoxetine, paroxetine, norfluoxetine, gemfibrozil, josamycin, triclosan, and triclocarban	Biosolids and soils	SPE	LC-ESI- MS/MS	C8 column (100 × 4.6 mm, 3 μm) MP: 0.1% formic acid (A) and Acetonitrile (B) Flow rate = 0.3 mL/min	30 min	Recovery = 26–146% LOQ = 0.1–27 ng/g R^2^ > 0.99	[46]
Quetiapine, fumarate, aripiprazole, and oxybenzone.	Sludge	SPE Oasis HLB	LC-ESI- MS/MS	C8 column (150 × 2.1 mm, 3 μm) MP: 0.1% formic acid in water (A) and methanol (B)	N.R.	Recovery = 100 ± 30% LOQ = 0.5–20 ng/L R^2^ ≥ 0.99	[42]
Triclosan	Sediment	SPE	GC-MS	HP-5 MS capillary column (30 m × 0.32 mm × 0.25 μm) Flow rate = 2.25 mL/min Temperature program: 50 °C/min for 1 min, 30 °C/min to 200 °C, then 2 °C/min to 230 °C, then 15 °C/min to 320 °C (10 min hold)	11 min	Recovery = 84.6 ± 5.7% LOD = 1.5 ng/g R^2^ = 0.999	[43]
Sulfadiazine, sulfapyridine, sulfathiazole, trimethoprim, sulfamerazine, sulfamethazine, sulfamethoxazole, naproxen, paracetamol, diclofenac acid, ibuprofen, ketoprofen, flumequine, ciprofloxacin, norfloxacin, ofloxacin, macrolides, clarithromycin, roxithromycin, erythromycin, tetracyclines, oxytetracycline, isochlortetracycline, tetracycline, cephalexin monohydrate, cefotaxime sodium, penicillin G, amphenicols, chloramphenicol, thiamphenicol, triclocarban, triclosan, carbamazepine, aminoglycoside, spectinomycin, antihyperlipidemic, gemfibrozil, diltiazem, diphenhydramine, and bisphenol A.	Sediment	SPE Oasis HLB	LC-ESI-MS/MS	C18 column (50 mm × 2.1 mm, particle size 1.8 μm). MP: Water (A) and Acetonitril (B) fro −ESI and 0.1% formic acid in water (A) and acetonitrile (B) for +ESI. Flow rate = 0.25 mL/min	N.R.	Recovery = 75–118% LOD = 0.01–0.6 ng/g LOQ = 0.03–1.7 ng/g R^2^ = 0.9937–0.9999	[86]
Ibuprofen, diclofenac, ketoprofen, bezafibrate, benzophenone 1, 2, and 4, methylparaben, morphine, and nomorphine	Sludge	MAE	LC-ESI-MS/MS	Separation of acidic analytes: C18 column (150 mm × 1.0 mm, particle size 1.7 μm). MP: (80:20) Water:Methanol conatin 1 mM Ammonium fluoride (A) and (5:95) Water:Methanol conatin 1 mM Ammonium fluoride (B) Flow rate = 0.04 mL/min Separation of basicanalytes: CHIRALPAK CBH HPLC column. MP: 1 mM ammonium acetate/methanol 85:15 (*v*/*v*) (Isocratic). Flow rate = 0.1 mL/min	N.R.	Recovery = 40.8–119.4% LOQ = 0.36–13.22 ng/g	[87]
Azithromycin, clarithromycin, metronidazole, ciprofloxacin, norfloxacin, vancomycin, and sulfamethoxazole	Sewage sludge	QuEChERS	LC- coupled to hybrid MS/MS/Ion trap	C18 column (100 mm × 4.6 mm, particle size 2.6 μm). MP: 0.2% formic acid (A) and 0.2% formic acid in acetonitrile (B) Flow rate = 0.5 mL/min	35 min	LOD = 2.2–490 ng/g LOQ = 7.4–1670 ng/g	[88]
Carbamazepine, triclosan, ibuprofen, and galaxolide	Sewage sludge	SPE Oasis HLB	LC-ESI-MS/MS	C18 column (100 mm × 2.1 mm, particle size 1.7 μm). MP: 0.1% formic acid in water (A) and 0.1% formic acid in methanol (B) Flow rate = 0.3 mL/min	16 min	Recovery = 89.7–93.6% LOQ = 0.6–1.2 ng/g R^2^ > 0.99	[94]
Telmisartan, airtazapine, amitriptyline, caffeine, carbamazepine, cetirizine, citalopram, venlafaxine, and triclosan.	Sewage sludge	Accelerated solvent extractor	LC-MS/MS	C18 column (100 mm × 3 mm, particle size 2.7 m). MP: 0.5 mM ammonium fluoride in water with 0.01% formic acid (A) and methanol (B) Flow rate = 0.4 mL/min	23.5 min	LOQ = 300–600 ng/g	[95]
Tetracycline, sulfamethoxazole, and triclocarban	Sediment	SPE Oasis HLB	LC-ESI-MS/MS	C18 column (100 mm × 2.1 mm, particle size 1.7 m). MP: Water (A) and 0.1% formic acid (B) Flow rate = 0.2 mL/min	7.5 min	Recovery = 81.3–100% LOD = 2–3 ng/g LOQ = 18.2–27.8 ng/g	[96]
Carbamazepin, ibuprofen, caffeine, sulfamethoxazole, methylparaben, and propylparaben,	Soil	Ultrasonic-assisted extraction and dispersive SPE	LC-ESI-MS/MS	C18 column (50 mm × 3 mm, particle size 2.6 μm). MP: 0.1% formic acid (A) and 0.1% formic acid in methanol (B) Flow rate = 0.6 mL/min	30 min	Recovery = 26–102% LOD = 0.07–1.34 ng/g LOQ = 0.24–4.46 ng/g	[97,98]

MP: mobile phase; N.R.: not reported; LOD: limit of detection; LOQ: limit of quantification; λ: wavelength; ESI: Electrospray ionization; MAE: microwave-assisted extraction.

## Data Availability

No new data were created or analyzed in this study.
